# Combining Bayesian genetic clustering and ecological niche modeling: Insights into wolf intraspecific genetic structure

**DOI:** 10.1002/ece3.4594

**Published:** 2018-10-30

**Authors:** Pietro Milanesi, Romolo Caniglia, Elena Fabbri, Felice Puopolo, Marco Galaverni, Rolf Holderegger

**Affiliations:** ^1^ Swiss Ornithological Institute Sempach Switzerland; ^2^ Area per la Genetica della Conservazione Istituto Superiore per la Protezione e la Ricerca Ambientale (ISPRA) Bologna Italy; ^3^ Department of Biology University of Naples Naples Italy; ^4^ Area Conservazione WWF Italia Rome Italy; ^5^ WSL Swiss Federal Research Institute Birmensdorf Switzerland; ^6^ Department of Environmental Systems Sciences ETH Zürich Zürich Switzerland

**Keywords:** *Canis lupus*, genetic clustering, human disturbance, landscape genetics, mixed‐effects models, *n*‐dimensional hypervolume

## Abstract

The distribution of intraspecific genetic variation and how it relates to environmental factors is of increasing interest to researchers in macroecology and biogeography. Recent studies investigated the relationships between the environment and patterns of intraspecific genetic variation across species ranges but only few rigorously tested the relation between genetic groups and their ecological niches. We quantified the relationship of genetic differentiation (*F*_ST_) and the overlap of ecological niches (as measured by *n*‐dimensional hypervolumes) among genetic groups resulting from spatial Bayesian genetic clustering in the wolf (*Canis lupus*) in the Italian peninsula. Within the Italian wolf population, four genetic clusters were detected, and these clusters showed different ecological niches. Moreover, different wolf clusters were significantly related to differences in land cover and human disturbance features. Such differences in the ecological niches of genetic clusters should be interpreted in light of neutral processes that hinder movement, dispersal, and gene flow among the genetic clusters, in order to not prematurely assume any selective or adaptive processes. In the present study, we found that both the plasticity of wolves—a habitat generalist—to cope with different environmental conditions and the occurrence of barriers that limit gene flow lead to the formation of genetic intraspecific genetic clusters and their distinct ecological niches.

## 1 INTRODUCTION

Genetic variation is key for the long‐term persistence and survival of species and populations. Its distribution across species ranges can reveal intraspecific information that is not necessarily represented in simple species occurrence patterns (Harrisson et al., 2016; Kovach et al., 2015). For instance, understanding the distribution of intraspecific genetic variation and how it relates to environmental factors can assist conservation management to assess species responses to environmental conditions and global change (Thomassen et al., 2010).

Recently, Gotelli and Stanton‐Geddes (2015) proposed the use of ecological niche models (ENMs), also known as habitat suitability models or species distribution models, for intraspecific genetic groups identified by Bayesian clustering, in order to account for the genetic diversity occurring within species. ENMs are widely used in various fields of ecology, evolution, biogeography, conservation biology, and landscape genetics (Beaumont et al., 2009; Guisan et al., 2013; Milanesi, Holderegger, Caniglia, Fabbri, & Randi, 2016; Milanesi, Breiner, Puopolo, & Holderegger, 2017; Milanesi, Holderegger, Bollmann, Gugerli, & Zellweger, Spear, Balkenhol, Fortin, McRae, & Scribner, 2010; Wang, Yang, Bridgman, & Lin, 2008). Gotelli and Stanton‐Geddes (2015) suggested that ENMs should be run for each intraspecific genetic cluster separately to provide insight into macroecological patterns caused by the heterogeneous evolutionary history across species’ distribution ranges (Marcer, Méndez‐Vigo, Alonso‐Blanco, & Picó, 2016). Species often display adaptation to particular combinations of environmental conditions in different parts of their whole range (Keller, Holderegger, & Strien, 2013). It is important to note that the approach of Gotelli and Stanton‐Geddes (2015) relies on simple correlations between genetic clusters and environmental factors. Thus, linking genetic patterns within species with ENMs does not necessarily assume that there is a causal effect between environmental factors and intraspecific genetic variation in terms of adaptation. In addition, most genetic markers (microsatellites, SSRs, or single‐nucleotide polymorphism, SNPs) used in genetic clustering are selectively neutral (Holderegger, Kamm, & Gugerli, 2006; Rellstab, Gugerli, Eckert, Hancock, & Holderegger, 2015). In summary, genetic clusters could either be caused by selective processes, caused by different environmental conditions (an adaptive process), or simply caused by restricted gene flow among clusters occurring in areas with different ecological conditions (a neutral process).

The approach of Gotelli and Stanton‐Geddes (2015) has so far only been applied in handful of studies (Harrisson et al., 2016; Ikeda et al., 2017; Marcer et al., 2016; Shinneman, Means, Potter, & Hipkins, 2016) , and the relationship between genetic distances among genetically defined clusters (e.g., genetic differentiation *F*_ST_; Dupanloup, Schneider, & Excoffier, 2002; Jombart, Devillard, Dufour, & Pontier, 2008) and distances or similarities among their ecological niches (e.g., niche overlap) has not been tested so far. Moreover, none of these studies specifically investigated the environmental factors differing among genetic clusters. However, in cases of low ecological niche overlap among intraspecific genetic clusters—that is, clusters showing cluster‐specific ecological niches—a given species could be considered as an assemblage of genetic or evolutionary lineages differing in their spatial distribution and their environments (Marcer et al., 2016).

Thus, in this study, we (a) estimated the pairwise ecological niche overlap between genetic groups resulting from spatial Bayesian clustering, (b) determined the relationship between genetic distances (*F*_ST_) and ecological niche overlap between identified genetic clusters, and (c) identified the ecological factors related to the spatial location of genetic clusters. For this purpose, we used a genetic data set on wolves (*Canis lupus*), which were molecularly identified during multiyear large‐scale monitoring projects based on carcasses, live‐trapped individuals, and non‐invasively collected samples from the central Apennines to the western Alps in Italy (Caniglia et al., 2012, 2013 ; Caniglia, Fabbri, Galaverni, Milanesi, & Randi, 2014; Fabbri et al., 2007). The population of wolves in Italy experienced a demographic bottleneck in the 1970s (Zimen & Boitani, 1975), which reversed in the 1980s due to legal protection, the occurrence of ample habitat in diverse landscapes abandoned by humans and the abundant availability of wild prey (Meriggi, Brangi, Schenone, Signorelli, & Milanesi, 2011). In about 40 years, the wolf population re‐expanded from south‐central Italy to the entire Apennine chain (including adjacent lower hills and plains) and the Italian and French western Alps (Caniglia et al., 2013; Fabbri et al., 2007; Galaverni, Caniglia, Fabbri, Milanesi, & Randi, 2016).

## 2 METHODS

### 2.1 Study area

**Figure 1:**
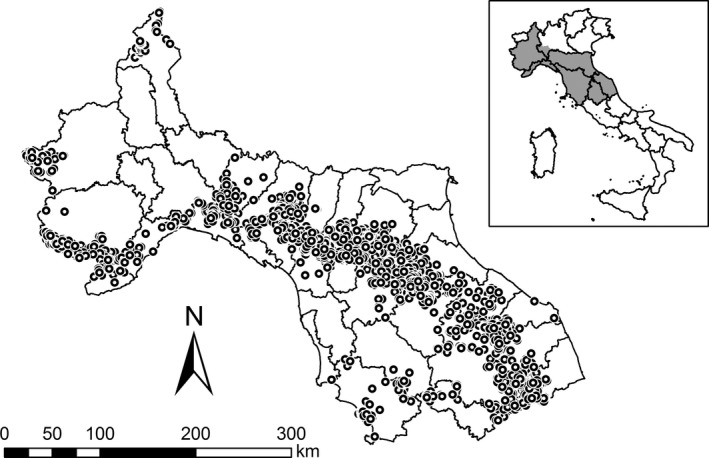
Study area in Italy (black lines indicate provincial and regional borders) and wolf sampling locations (white dots with black circles)

Data collection was carried out from the central Apennines to the western Alps in Italy, in a study area of 97,044 km^2^ (6°62′–13°91′E; 46°46′–42°39′N; Figure 1). The study area was characterized by a large altitudinal range (0–4,634 m a.s.l.), distinct climatic gradients (from temperate to alpine), and diverse human land uses. It was characterized by a high diversity of habitats, such as different types of meadows, pastures, rocky surfaces, and even glaciers in high mountains. In the lower mountains and foothills, abandoned fields currently changing into semi‐natural shrublands and deciduous, mixed or evergreen forests were most abundant. In the valleys, plains, and at the coast, agricultural fields and urban areas were dominant.

### 2.2 Genetic data set

Putative wolf samples (mainly feces but also blood or muscular tissues from carcasses as well as saliva, urine, and hairs; *N* = 9,317) were collected by more than 400 trained operators (Italian State Forestry Corps, park rangers and technicians, wildlife managers, researchers, students, and volunteers). Training was done by wolf experts, and operators were instructed to collect samples as fresh‐looking as possible, excluding degraded ones. Samples were collected along randomly chosen trails and country roads across all habitat types, at least once per season. Sampling was part of a long‐term monitoring program on wolves in Italy (from 2000 to 2011; Caniglia et al., 2012, 2014 ; Fabbri et al., 2007). Samples were stored at −20°C in 10 volumes of 95% ethanol or Tris/SDS buffer (Caniglia et al., 2012). The MULTIPROBE II^EX^ Robotic Liquid Handling System of Perkin Elmer and the QIAGEN stool and tissue extraction kits were used to extract DNA. Multi‐locus genotypes, gender, and taxon (i.e., wolf, dog, or hybrid) were determined using 12 unlinked neutral microsatellites, a sex‐specific restriction site and taxon‐specific markers following the methods described in Caniglia et al., (2014, 2013 . PCR amplification of each sample was carried out four to eight times. A total of 3,815 samples were reliable genotyped at all markers and belonged to 923 unrelated wolf individuals (Figure 1), 93 dogs and 118 wolf ×dog hybrids (Milanesi et al., 2016, Milanesi, Holderegger, Bollmann, et al., ).

### 2.3 Genetic clustering

To identify genetic clusters of Italian wolves, we considered the 923 wolf individuals and used the Bayesian genetic clustering method implemented in TESS 2.3 (Chen, Durand, Forbes, & François, 2007), incorporating spatial information on sampling locations (considering the spatial coordinates of the location where a given wolf has been sampled for the first time). We used the admixture model to calculate 10 runs for *K*_max_ = 2–6 clusters (500,000 sweeps, 100,000 burn‐in) using three different values of the spatial interaction parameter *h* (0, 0.6—the default value—and 0.99 indicating no spatial, medium, and high spatial dependence, respectively).

The optimal number of clusters was estimated through the deviance information criterion (DIC) plot (Chen et al., 2007). For the most likely number of clusters, mean cluster membership probabilities of the 10 runs were estimated with CLUMPP 1.1.2 (Jakobsson & Rosenberg, 2007) and average cluster membership probabilities were then interpolated across the whole study area by thin plate spline function (Tps) in the R package FIELDS (v. 3.3.1; Furrer, Nychka, & Sain, 2009; again only considering the spatial coordinates of the location where a given wolf has been sampled for the first time).

We estimated genetic differentiation between the identified genetic clusters with pairwise *F*_st_‐values calculated in GENALEX (9,999 permutations; Peakall & Smouse, 2006).

### 2.4 Predictor variables

We collected a total of 13 predictor variables encompassing environmental, topographic, and anthropogenic factors. Specifically, we estimated habitat diversity and the percentages of six land cover types derived from CORINE Land Cover 2006 IV Level, with a minimum mapping unit/width of 25 ha/100 m and a thematic accuracy ≥85% (https://www.sinanet.isprambiente.it/it/sia-ispra/download-mais/corine-land-cover/corine-land-cover-2006-iv-livello/view; Table 1). From a digital elevation model of Italy (20 m spatial resolution; https://www.sinanet.isprambiente.it/it/sia-ispra/download-mais/dem20/), we obtained the topographic variables altitude, slope, and landscape roughness (Table 1). We also considered human population density (at a spatial resolution of 1 km; https://ec.europa.eu/eurostat/web/gisco/geodata/reference-data/population-distribution-demography) as well as the presence and distance from human settlements (i.e., urban areas, villages, derived from CORINE Land Cover 2006 IV Level).

**Table 1:** Environmental factors used in generalized linear models

Feature	Variable	Units	VIF
Land cover	Coniferous forests	Percentage (%)	1.128
	Mixed woods	Percentage (%)	1.101
	Shrublands	Percentage (%)	1.188
	Deciduous forests	Percentage (%)	1.337
	Meadows	Percentage (%)	1.551
	Cultivated fields	Percentage (%)	> 3
	Shannon index of habitat diversity	Sum of natural logarithm of each category proportion in the sample grid	1.301
Topography	Altitude	Meter a.s.l. (m)	2.486
	Slope	Degree (°)	>3
	Landscape roughness	Ratio of the average length of isolines in the sample grid over sample grid side length	>3
Anthropogenic factors	Human population density	Number per km^2^	1.198
	Human settlements	Percentage (%)	1.313
	Distance from human settlements	Meter (m)	1.791

Note

Variables with a variance inflation factor (VIF) >3 were removed from further analysis due to multi‐collinearity with other variables.

We calculated the variance inflation factor (VIF) for all the variables and removed predictor variables with values higher than 3 (i.e., highly correlated to other predictors; Zuur, Ieno, & Elphick, 2010; Table 1) to avoid multi‐collinearity among predictors. Specifically, we removed cultivated fields, slope, and landscape roughness, because they all exhibited a VIF >3, and only considered the remaining 10 variables in further analyses (Table 1). We also estimated spatial autocorrelation within each predictor variable through Moran's *I* index (Table S1) using the R package RASTER (v. 2.5‐8. Hijmans, 2011). We found relatively high and positive Moran's *I* values considering the entire study area (indicating positive spatial autocorrelation, e.g., clustering; Cliff & Ord, 1981) but low values (close to zero, indicating a random pattern with no spatial autocorrelation; Cliff & Ord, 1981) considering only the values of the predictor variables at the locations were wolves where sampled (Supporting Information Table S1).

### 2.5 Relating genetic differentiation and ecological niche overlap

We estimated the ecological niche of each genetic cluster considering the locations of wolves belonging to a particular cluster by *n*‐dimensional hypervolumes (Blonder, Lamanna, Violle, & Enquist, 2014; Blonder, Lamanna, Violle, & Enquist, 2017; see Supporting Information Table S2 for details on model parameters), which, in contrast to other ENMs, allows estimation of the ecological hyperspace considering the whole set of predictors (Blonder et al., 2014). We quantified pairwise ecological niche overlap between clusters of wolves as the intersection of two shared/summed ecological niches in the hyperspace. We performed these analyses in the R package HYPERVOLUME (v. 2.0.8; Blonder et al., 2014). We also provided minimum, mean, and maximum values of the predictor variables of the hypervolume in the minimum convex polygon estimated for each wolf cluster (Supporting Information Table S3).

We then compared the pairwise *F*_st_‐values between genetic clusters (response variable) and the corresponding ecological niche overlap between two clusters (fixed effect) in a linear mixed‐effects model (a Toeplitz covariance matrix was considered as random effect to account for the non‐independence of distance measurements between pairs of clusters; Milanesi et al., 2016; Milanesi, Breiner, et al., 2017; Milanesi, Holderegger, Bollmann, et al., ; Selkoe et al., 2010; Van Strien, Keller, & Holderegger, 2012). Actually, linear mixed‐effects models have recently been identified as the best performing among several landscape genetics technics (Shirk, Landguth, & Cushman, 2018). Specifically, our approach is conceptually similar to an isolation by environment test (Wang & Bradburd, 2014; Wang, Glor, & Losos, 2013), but instead of relate pairwise genetic and environmental distances (often derived by least‐cost paths) among populations, we related pairwise genetic distances with ecological niche overlap between clusters. In our approach, high standardized regression coefficient (*β*) values correspond to a large presumed effect of the predictor on the response variable. We expected an inverse relationship between *F*st‐values and ecological niche overlap (and thus a negative standardized *β* value), as genetic differentiation should decrease with increasing niche overlap. We used 10,000 permutations to assess significance of linear mixed‐effects models using the PGIRMESS package (v. 1.6.7; Giraudoux, 2013) in R.

### 2.6 Relating cluster distribution and predictor variables

To identify the particular environmental factors related to the spatial distribution of the different genetic clusters of wolves in Italy, we developed spatial conditional autoregressive models (CAR) developed using the SPDEP package in R (v. 0.6‐15; Bivand et al., 2011), which consider autocorrelation among locations (while low at our wolf locations; Supporting Information Table S1), using the cluster membership of each individual wolf as dependent variable. To identify the best model(s) per cluster, we applied an information theoretic approach (Burnham & Anderson, 2004) through model selection and multi‐model inference (testing all possible combinations of predictor variables). Specifically, the Akaike information criterion (AIC; Akaike, 1974) was used, and models were ranked based on ΔAIC (considering only models with ΔAIC < 2; Burnham & Anderson, 2004). We estimated standardized regression *β*‐coefficients (and their standard errors) as well as significance and importance (calculated as the sum of the Akaike weights, *W*) of all the predictor variables entered in the best model(s) per genetic cluster through model averaging. We carried out these analyses using the R package MUMIN (v. 1.0.0. Barton, 2009). We also calculated sampling effort through Gaussian kernel density (Elith, Kearney, & Phillips, 2010) based on all sampling locations (i.e., including wolves, dogs, and hybrids locations) and used the resulting values as case weights in the above‐mentioned CAR models.

## 3 RESULTS

**Figure 2:**
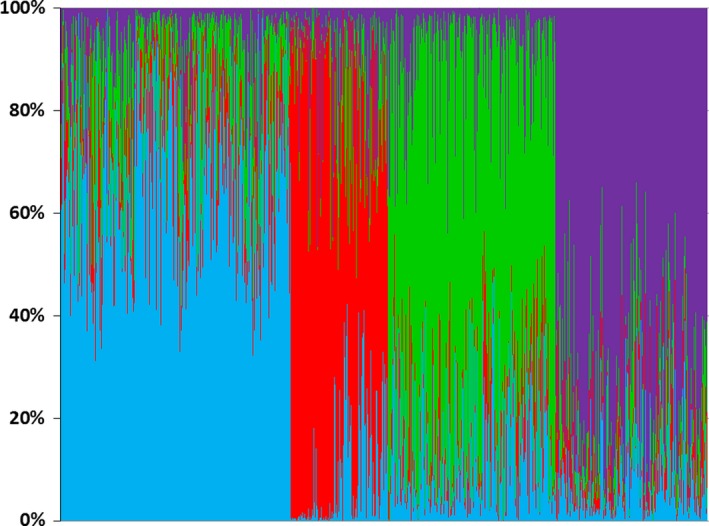
Bayesian clustering analysis showing barplots for the proportion of cluster memberships assigned to individual wolves under the estimated optimal numbers of clusters (*K*_max_ **=* *4). Different colors indicate different clusters

**Figure 3:**
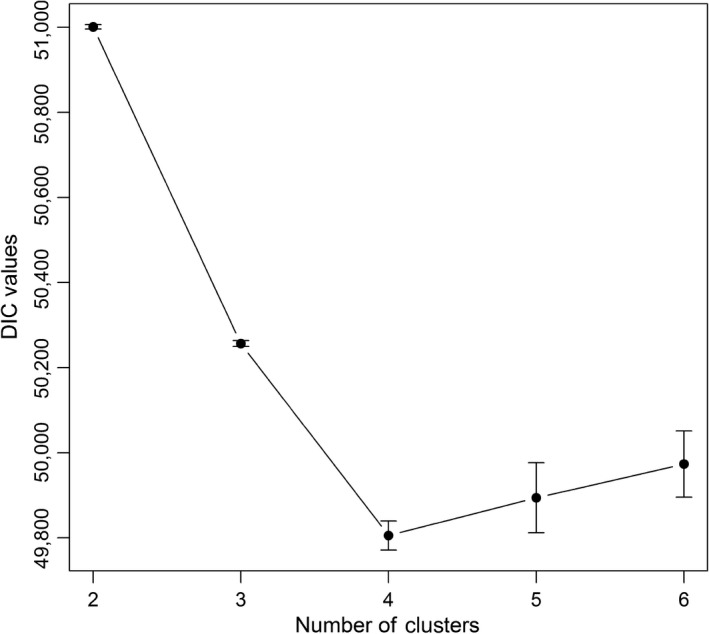
Estimation of the number of genetic clusters (*K*_max_ = 2–6) of wolves in Italy based on the mean (±95% confidence intervals from 10 runs) deviance information criterion (DIC)

**Figure 4:**
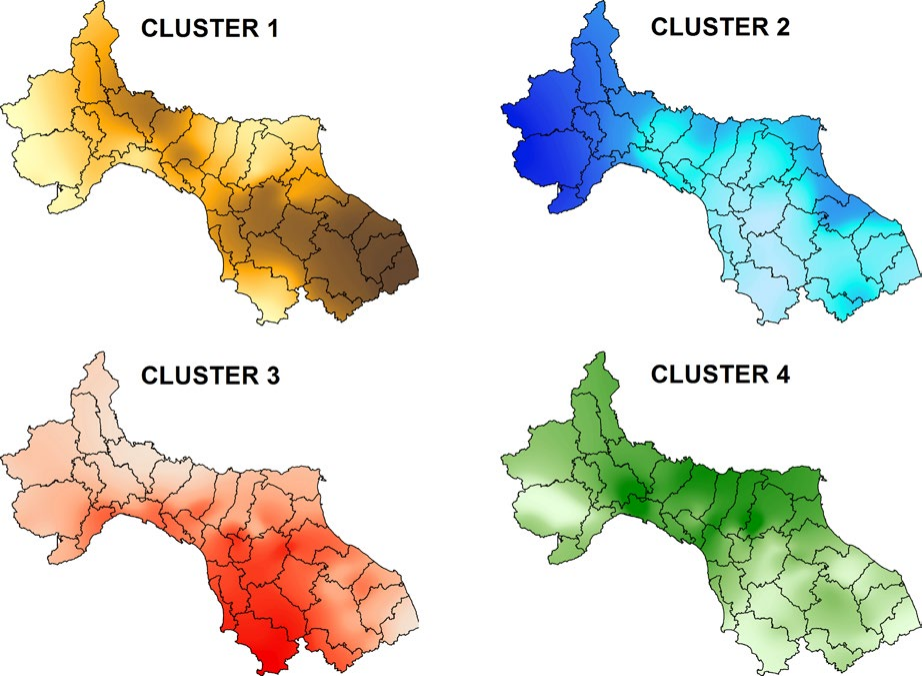
Genetic clusters of wolves in Italy. Interpolation maps of membership coefficients of four genetic clusters of wolves are shown (dark to light color shadings indicates higher to lower cluster membership probabilities)

Bayesian genetic cluster analysis suggested the existence of four genetic clusters (Figure 2), as indicated by the lowest DIC value at *K*_max_ = 4 (49,805.75; 95% confidence interval: ±34.15; Figure 3) in the DIC plot (Figure 2). These four clusters were consistent across the three different spatial interaction factors (*h*) tested. We thus based further analyses on the default spatial interaction factor of *h* = 0.6 and the resulting four genetic clusters. The highest number of wolf individuals was recorded in cluster 1 (*N* = 329), followed by cluster 3 (*N* = 239), cluster 4 (*N* = 217), and cluster 2 (*N* = 138). Cluster 1 was located on the eastern side of the central Apennines (EC hereafter), cluster 2 occurred in the western Alps (WA), cluster 3 on western side of the central Apennines (WC), and cluster 4 in the northern Apennines (NA; Figure 4).

Pairwise comparisons showed statistically significant (*p* < 0.001) genetic differentiation among clusters. Ecological niche overlap between clusters was moderate, with values ranging between 0.361 and 0.451 (Table 2). These are relative values (ranging from 0 to 1), and hence, the overlap is somewhere in the middle between low and high.

**Table 2:** Pairwise genetic differentiation *F*st (below diagonal) and pairwise ecological niche overlap (above diagonal) between clusters of wolves

	Eastern‐central Apennines (EC)	Western Alps (WA)	Western‐central Apennines (WC)	Northern Apennines (NA)
EC	–	0.438	0.361	0.415
WA	0.031*	–	0.428	0.451
WC	0.055*	0.042*	–	0.401
NA	0.033*	0.018*	0.013*	–

^*^ *p* < 0.001.

Linear mixed‐effects models with pairwise genetic differentiation *F*_ST_ as dependent variable showed a significant relationship (*p* < 0.001) with ecological niche overlap between clusters with a standardized β‐coefficient of 0.557.

Considering EC, the best models (*N* = 11; AIC ranging between 995.43 and 997.42, better than a random model at AIC = 1,029.28) included altitude, meadows, and habitat diversity, which were all positively related to cluster membership and had the highest W and *p* < 0.001 (Table 3), while deciduous and coniferous forests, human population density, shrublands, mixed woods, and human settlements showed lower values of *W* and were non‐significant (Table 3). The best models of WA (*N* = 9; AIC ranging between 278.41 and 280.40, better than a random model at AIC = 300.67) included altitude, shrublands, coniferous, and deciduous forests (only the latter with negative *β* value) with the highest *W* and *p* < 0.001 (Table 3), while habitat diversity, mixed woods, meadows, human settlements, and human population density showed lower values of *W* and were non‐significant (Table 3). In contrast, the best models of WC (*N* = 16; AIC ranging between 933.04 and 935.04, better than a random model at AIC = 1,018.07) included deciduous forests and mixed woods, both positively related to cluster membership, with the highest *W* and *p* < 0.001 (Table 3), while human settlements, coniferous forests, altitude, human population density, habitat diversity, meadows, and shrublands density showed lower values of *W* and were non‐significant (Table 3). Finally, in the best models of NA (*N* = 12; AIC ranging between 680.43 and 682.42, better than a random model at AIC = 701.18) altitude, deciduous forests, and meadows (only the latter with negative *β* value) showed the highest *W* and *p* < 0.001 (Table 3), while habitat diversity, human settlements, mixed woods, coniferous forests, shrublands, and human population density showed lower values of *W* and were non‐significant (Table 3).

**Table 3:** Average standardized coefficients (*β*), standard errors (*SE*), *p*‐values (*p*) and relative importance from Akaike weights (*W*) based on averaging spatial conditional autoregressive model (CAR) for cluster membership (considering only models with ΔAIC < 2)

Cluster	Variable	*β*	*SE*	*p*	*W*
EC	(Intercept)	−1.33	0.35	<0.001	–
	Altitude	0.64	0.01	<0.001	0.99
	Meadows	0.71	0.01	<0.001	0.99
	Shannon index of habitat diversity	0.92	0.24	<0.001	0.99
	Deciduous forests	0.01	0.00	0.361	0.15
	Human population density	−0.01	0.00	0.471	0.13
	Coniferous forests	0.01	0.01	0.550	0.12
	Shrublands	0.01	0.00	0.586	0.12
	Mixed woods	0.01	0.00	0.729	0.11
	Human settlements	−0.01	0.01	0.800	0.10
WA	(Intercept)	−2.84	0.42	<0.001	–
	Altitude	0.91	0.01	<0.001	0.99
	Shrublands	0.77	0.02	<0.001	0.99
	Coniferous forests	0.89	0.02	<0.001	0.99
	Deciduous forests	−0.61	0.01	<0.001	0.99
	Shannon index of habitat diversity	−0.33	0.36	0.364	0.18
	Mixed woods	0.01	0.01	0.496	0.15
	Meadows	0.01	0.00	0.659	0.13
	Human settlements	−0.01	0.02	0.788	0.12
	Human population density	−0.01	0.00	0.887	0.12
WC	(Intercept)	−1.45	0.28	<0.001	–
	Deciduous forests	0.93	0.01	<0.001	0.99
	Mixed woods	0.86	0.01	<0.001	0.99
	Human settlements	−0.02	0.01	0.071	0.83
	Coniferous forests	−0.01	0.01	0.258	0.36
	Altitude	0.01	0.00	0.225	0.29
	Human population density	−0.01	0.00	0.339	0.18
	Shannon index of habitat diversity	0.23	0.27	0.390	0.15
	Meadows	0.01	0.00	0.362	0.11
	Shrublands	0.01	0.01	0.689	0.05
NA	(Intercept)	−0.39	0.47	0.412	–
	Altitude	0.84	0.01	<0.001	0.99
	Deciduous forests	0.91	0.01	<0.001	0.99
	Meadows	−0.62	0.02	<0.001	0.99
	Shannon index of habitat diversity	0.49	0.29	0.090	0.73
	Human settlements	−0.02	0.01	0.160	0.67
	Mixed woods	−0.01	0.01	0.211	0.39
	Coniferous forests	−0.01	0.01	0.285	0.30
	Shrublands	−0.01	0.01	0.288	0.24
	Human population density	−0.01	0.00	0.969	0.08

## 4 DISCUSSION

In this study, through the analysis of neutral genetic markers (i.e., microsatellites), we identified four genetic clusters of wolves in Italy and developed cluster‐specific ENMs to estimate niche overlap between them. We found moderate ecological niche overlap between the resulting clusters, which was nevertheless significantly related to the genetic distances between them, suggesting that genetically different clusters live in different environmental conditions. Accordingly, we identified cluster‐specific combinations of environmental factors influencing wolf occurrence in Italy.

### 4.1 Genetic clusters of wolves in Italy

Why should genetic clusters reflect differences in ecological niches among clusters? There are two main possible explanations: (a) genetic clusters might reflect differential adaptation to different environments (as detected by ENMs) caused by selection; (b) genetic clusters occupy different ecological niches, but the genetic differences among them are caused by restricted gene flow. Though the two processes are different in nature, they are not mutually exclusive (Hoffmann & Willi, 2008).

Genetic clusters are often identified based on a small number of microsatellite (SSRs) markers or a larger number of SNPs. While the majority of SSRs is neutral by definition and is usually not linked to adaptive regions within the genome of a study species, a smaller or larger proportion of SNPs (usually about 5%; Schoville et al., 2012) is of adaptive relevance (i.e., is located in adaptive genes or is closely linked to adaptive regions). In both cases, most or all of the genetic markers used for genetic clustering behave neutrally, suggesting that there probably is no direct adaptive relationship between genetic clusters and the ecological niches they occupy. In fact, demographic spatial patterns can mimic and confound adaptive signals (Beaumont & Nichols, 1996; Foll & Gaggiotti, 2008; Holderegger et al., 2008; Rellstab et al., 2015).

If so, intraspecific genetic clusters can also be caused by limited movement, dispersal, and gene flow among different regions within the distribution range of a species (Gotelli & Stanton‐Geddes, 2015), and the genetic differentiation among them is then affected by the interplay of gene flow and genetic drift (Hutchison & Templeton, 1999; Slatkin, 1987). Restricted gene flow among clusters could be caused by many factors affecting movement and dispersal, especially by geographical or landscape barriers—a topic widely explored in evolution, biogeography, landscape ecology, and landscape genetics (Andrew, Ostevik, Ebert, & Rieseberg, 2012; Holderegger & Wagner, 2008; Manel, Schwartz, Luikart, & Taberlet, 2003; McCairns & Bernatchez, 2008; Storfer et al., 2007; Storfer, Murphy, Spear, Holderegger, & Waits, 2010; Temple, Hoffman, & Amos, 2006). Especially in mammals, intrinsic factors such as behavior or the specific use of food resources could also cause limited movement and dispersal among clusters (Pilot et al., 2006; Vonholdt et al., 2010).

The four genetic clusters identified in this study largely coincided with the topology of the main mountain regions occupied by wolves in Italy (parts of the Apennines and the Alps; Figure 3, Supporting Information Figure S1), occupying four continuous areas located on the eastern and western sides of the central Apennines, in the northern Apennines and in the western Alps (Figure 3). A previous study on the history and dynamics of the wolf recolonization in the Apennines detected only three clusters in Italy, namely the central Apennines, the northern Apennines and the western Alps (Fabbri et al., 2007), based on a different genetic data set than the present one. Thus, an interesting result of our analyses was the splitting of the central Apennine wolf population into two distinct genetic clusters, one on the western and one on the eastern slope of the Apennines (Figure 3). The discovery of another cluster in the Italian wolf population was rather unexpected due to the assumed high mobility of wolves. However, the two separated genetic clusters of wolves on opposite slopes of the Apennines could be the result of recent re‐colonization of low‐mountain or hilly areas close to the Tyrrhenian and the Adriatic Sea, originating from the former core habitat of wolves at higher altitudes in the central Apennines (Caniglia et al., 2013; Galaverni et al., 2017; Giacchini, Scotti, & Zabaglia, 2012). The cluster in the western Alps was mainly founded by long‐distance dispersal from the Apennines at the onset of the re‐colonization process of the Alps, followed by the setting of territorial behavior of reproducing packs there (Fabbri et al., 2007; Valière et al., 2003). Such a scenario is supported by similar patterns of genetic structure found for wild cats (*Felix silvestris*) in Italy (Mattucci et al., 2013).

### 4.2 Different niches of wolf clusters in Italy

The four identified clusters of wolves were related to different ecological factors. Specifically, we found significant and positive relationships between WC and mixed woods and deciduous forests, while WA was positively related to coniferous forests and NA was positively related to deciduous forests. Thus, our results highlighted the importance of woods and forests in providing suitable habitat and shelter for wolves (Bassi, Willis, Passilongo, Mattioli, & Apollonio, 2015; Jędrzejewski, Niedzialkowska, Mysłajek, Nowak, & Jędrzejewska, 2005; Llaneza, López‐Bao, & Sazatornil, 2012; Meriggi et al., 2011; Milanesi, Meriggi, & Merli, 2012). However, deciduous forests were negatively related to WA. Actually, the areas of occurrence of EC and NA were characterized by a low percentage of coniferous forests, but a high percentage of deciduous forests and mixed woods, while those of WA were mainly covered by coniferous forests. These results suggest that wolves simply are related to the main regional forest type, irrespective of its nature. Moreover, EC, WA, and NA were positively related to altitude, probably because of the abundance of prey, availability of safe areas, and lower disturbance by human activities at higher altitudes (Bassi et al., 2015; Glenz, Massolo, Kuonen, & Schlaepfer, 2001; Jędrzejewski et al., 2005; Llaneza et al., 2012; Pilot et al., 2006).

Shrublands were positively related to WA, probably because of high amounts of open shrublands consisting of land used as pastures by freely roaming livestock in the corresponding region (Eggermann, Costa, Guerra, Kirchner, & Petrucci‐Fonseca, 2011). Meadows were positively related to EC, as a large share of meadows in a region potentially translates into a high availability of livestock as prey. Thus, meadows become important for wolves, in regions where forests are sparse (Jędrzejewski et al., 2005). In contrast, meadows were negatively related to the distribution of NA due to small extension of meadows or low accessibility of livestock as prey in this area (Meriggi et al., 2011). EC was positively related to habitat diversity, because wolves tend to use different habitat types for different activities (Houle, Fortin, Dussault, Courtois, & Ouellet, 2010) and because habitat heterogeneity is a known driver of wild ungulate diversity and distribution (Cromsigt, Prins, & Olff, 2009). In summary, wolf clusters in Italy seem to be simply associated with the most abundant land cover types of their respective area, showing that high ecological flexibility of wolves enabling them to cope with diverse and even fragmented landscapes (Llaneza et al., 2012).

Genetic clusters of animals should also be affected by anthropogenic factors. For instance, animals could show avoidance behavior against human settlements or roads. Such avoidance has been assessed in several wild animals (Cushman, McKelvey, Hayden, & Schwartz, 2006) and is well known to also affect wolf occurrence (Gaillard et al., 2010). Indeed, we found that human disturbance, in terms of human population density and human settlements, tended to be related to genetic clusters in Italian wolves, but not in a consistent and statistically significant way. Nevertheless, in agreement with other studies (Jędrzejewski et al., 2005; Llaneza et al., 2012; Milanesi et al., 2016), the distribution of the four different clusters were negatively related to human population density and human settlements, while distance from human settlements was not included in any of the best models for all clusters.

### 4.3 Conclusions, management implications, and future research

In this study, we found that different genetic clusters of wolves in Italy are differently related to land cover types, probably caused by two main effects. First, the plasticity of the wolf—a distinct habitat generalist (Llaneza et al., 2012)—which allows it to adapt to diverse regions and landscapes with different environmental conditions. Second, the occurrence of geographical barriers limiting gene flow among wolf clusters. While the former can be inferred from the different relationships between different wolf clusters, and different environment factors, the latter is proved by wolf clusters being mainly separated by mountainous terrains. As long dispersal distances in wolves have been recorded—also across human‐dominated landscapes (Andersen et al., 2015)—wolf clusters in Italy are probably still linked by some gene flow (*F*_ST_ 0.055). In addition, the wolf is a territorial carnivore living in hierarchical packs and the occurrence of established packs can thus result in moving animals not settling down, because of aggressive behavior and competition of resident individuals, resulting in limited gene flow, finally contributing to genetic clustering in Italian wolves.

Finally, we remark that when using genetic clusters based on neutral loci in combination with ENMs, one pitfall is to simply interpret the results in the light of adaptation. However, there is no direct link between neutral and adaptive genetic diversity or differentiation (Merilä & Crnokrak, 2001; Reed & Frankham, 2001). Differences in the ecological niches of genetic clusters should thus be interpreted in light of neutral processes that influence movement, dispersal, and gene flow among genetic clusters (Gotelli & Stanton‐Geddes, 2015), as done in the present study. However, further studies should compare genetic clusters separately based on neutral and adaptive genetic markers and relate them to ecological niches (as identified by ENMs; Deagle, Jones, Absher, Kingsley, & Reimchen, 2013; Manel & Holderegger, 2013). Since adaptive markers do not have to be in Hardy–Weinberg equilibrium nor in linkage equilibrium, commonly used Bayesian genetic clustering techniques cannot be applied. Nevertheless, assumption‐free methods are now available and can be used for this purpose (e.g. TESS 3; Caye, Deist, Martins, Michel, & François, 2016). Environmental association analyses (linking potentially adaptive genes to particular environmental factors; Rellstab et al., 2015) can then be combined with ENMs to tests whether genetic clusters based on adaptive genes are related to different ecological factors. In such analyses, spatial coincidence of adaptive and neutral genetic clusters would make a strong case for adaptively relevant differences among genetic clusters.

## CONFLICT OF INTEREST

None declared.

## AUTHORS CONTRIBUTIONS

P.M. and R.H. designed the analyses; R.C., E.F., and M.G. produced the Italian canid database and shared ideas to realize the article; P.M. carried out analyses and wrote the manuscript; R.H. and F.P. shared in the writing of the manuscript. All authors approved the final version of the manuscript.

## DATA ACCESSIBILITY

Due to sensitive information (locations of an endangered species), the data of the present study cannot be made openly accessible.
