# Supplementary figures and images for: Combining Bayesian genetic clustering and ecological niche modeling: Insights into wolf intraspecific genetic structure
Source: Ecol Evol. 2018 Oct 30;8(22):11224–34. doi: 10.1002/ece3.4594 (PMC6262746; doi:10.1002/ece3.4594)

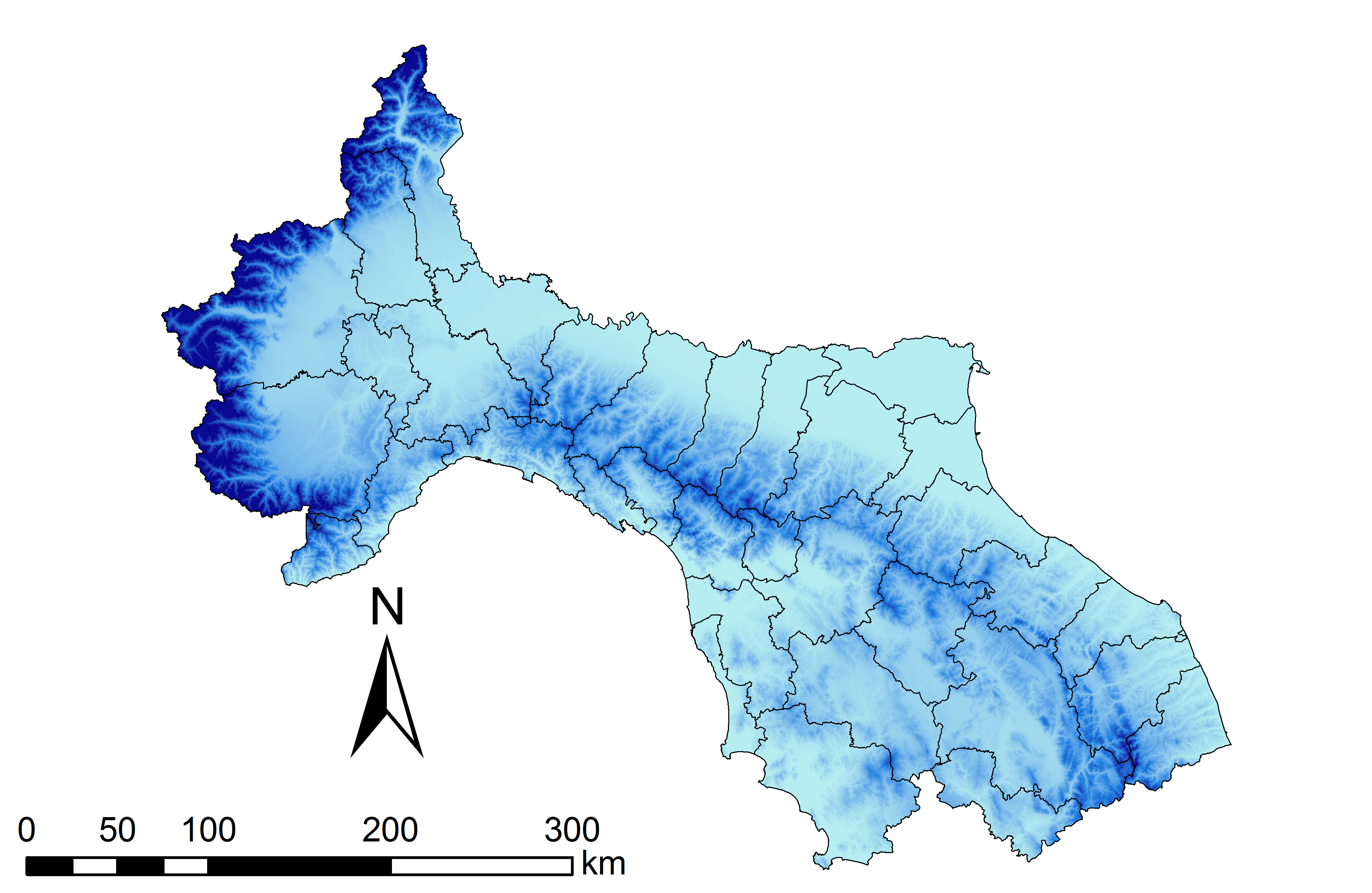

Supplement: Supplementary file 1 [file ECE3-8-11224-s001.tif]
