# Supplementary material for: Combining Bayesian genetic clustering and ecological niche modeling: Insights into wolf intraspecific genetic structure
Source: Ecol Evol. 2018 Oct 30;8(22):11224–34. doi: 10.1002/ece3.4594 (PMC6262746; doi:10.1002/ece3.4594)
Supplement: Supplementary file 2 [file ECE3-8-11224-s002.docx]

Table S1. Spatial autocorrelation of the predictor variables considering the whole study area and only the locations were wolves where sampled (Moran’s I values are shown). Values of Moran’s I range between 1 (indicating strong positive spatial autocorrelation, e.g. clustering) and 1 (indicating strong negative spatial autocorrelation, e.g. dispersion) while 0 indicates a random pattern with no spatial autocorrelation (Cliff & Ord, 1981).

| **Variable** | **Study area** | **Wolf locations** |
| --- | --- | --- |
| Altitude | 0.932 | 0.053 |
| Deciduous forests | 0.722 | 0.045 |
| Meadows | 0.572 | 0.031 |
| Coniferous forests | 0.518 | 0.062 |
| Human settlements | 0.508 | 0.003 |
| Mixed woods | 0.498 | 0.043 |
| Human population density | 0.423 | 0.006 |
| Shannon index of habitat diversity | 0.418 | 0.025 |
| Shrub-lands | 0.411 | 0.032 |

Cliff, A.D. & Ord, J.K. (1981) Spatial processes: models and applications, London. *Pion*, Pages 266.
