# Supplementary material for: Combining Bayesian genetic clustering and ecological niche modeling: Insights into wolf intraspecific genetic structure
Source: Ecol Evol. 2018 Oct 30;8(22):11224–34. doi: 10.1002/ece3.4594 (PMC6262746; doi:10.1002/ece3.4594)
Supplement: Supplementary file 3 [file ECE3-8-11224-s003.docx]

Table S2. Model parameters used to develop each genetic cluster *n*-dimensional hypervolume following Blonder *et al.* (2014; 2017).

| **Parameter** | **Value** |
| --- | --- |
| Predictor variables | Standardized according to their mean and standard deviation |
| Number of dimensions, *n* | 10, equal to the number of unrelated predictor variables considered |
| Bandwidth vector, *h* | Calculated from the data using Silverman’ estimator |
| Quantile threshold, *τ* | 0 equal to the fraction of the probability density excluded from the hypervolume |
| Number of random points around each location | 10,000 |
