# Supplementary material for: Combining Bayesian genetic clustering and ecological niche modeling: Insights into wolf intraspecific genetic structure
Source: Ecol Evol. 2018 Oct 30;8(22):11224–34. doi: 10.1002/ece3.4594 (PMC6262746; doi:10.1002/ece3.4594)
Supplement: Supplementary file 4 [file ECE3-8-11224-s004.docx]

Table S3. Minimum, mean and maximum values of the predictor variables in the minimum convex polygon around wolf occurrences estimated for each wolf cluster separately.

|  | EC | | | WA | | | WC | | | NA | | |
| --- | --- | --- | --- | --- | --- | --- | --- | --- | --- | --- | --- | --- |
|  | Min | Mean | Max | Min | Mean | Max | Min | Mean | Max | Min | Mean | Max |
| Altitude | 2.75 | 415.94 | 2213.56 | 0.00 | 369.06 | 4260.00 | 0.00 | 387.75 | 2000.88 | 24.25 | 582.25 | 2000.88 |
| Deciduous forests | 0.00 | 25.00 | 100.00 | 0.00 | 6.25 | 100.00 | 0.00 | 31.25 | 100.00 | 0.00 | 50.00 | 100.00 |
| Coniferous forests | 0.00 | 0.00 | 100.00 | 0.00 | 0.00 | 100.00 | 0.00 | 0.00 | 100.00 | 0.00 | 0.00 | 100.00 |
| Mixed woods | 0.00 | 0.00 | 100.00 | 0.00 | 0.00 | 100.00 | 0.00 | 0.00 | 100.00 | 0.00 | 0.00 | 100.00 |
| Shrub-lands | 0.00 | 0.00 | 100.00 | 0.00 | 0.00 | 100.00 | 0.00 | 0.00 | 100.00 | 0.00 | 0.00 | 100.00 |
| Meadows | 0.00 | 0.00 | 100.00 | 0.00 | 0.00 | 100.00 | 0.00 | 0.00 | 100.00 | 0.00 | 0.00 | 100.00 |
| Human settlements | 0.00 | 0.00 | 100.00 | 0.00 | 0.00 | 100.00 | 0.00 | 0.00 | 100.00 | 0.00 | 0.00 | 100.00 |
| Human population density | 0.00 | 21.00 | 5352.00 | 0.00 | 31.00 | 5180.00 | 0.00 | 22.00 | 5285.00 | 0.00 | 21.00 | 4746.00 |
| Shannon index of habitat diversity | 0.72 | 1.14 | 2.22 | 0.72 | 1.09 | 2.15 | 0.72 | 1.14 | 2.15 | 0.72 | 1.14 | 2.10 |
